# Killer Cell Immunoglobulin-Like Receptor Alleles Alter HIV Disease in Children

**DOI:** 10.1371/journal.pone.0151364

**Published:** 2016-03-16

**Authors:** Kumud K. Singh, Min Qin, Sean S. Brummel, Konstantia Angelidou, Rodney N. Trout, Terence Fenton, Stephen A. Spector

**Affiliations:** 1 Department of Pediatrics, University of California, San Diego, La Jolla, California, United States of America; 2 Center for Biostatistics in AIDS Research, Harvard T.H. Chan School of Public Health, Boston, Massachusetts, United States of America; 3 Rady Children’s Hospital, San Diego, California, United States of America; Emory University School of Medicine, UNITED STATES

## Abstract

**Background:**

HLA class I molecules are ligands for killer cell immunoglobin like receptors (KIR) that control the antiviral response of natural killer (NK) cells. However, the effects of *KIR* and *HLA* (*KIR/HLA*) alleles on HIV disease of children have not been studied.

**Methods:**

993 antiretroviral naïve children with symptomatic HIV infection from PACTG protocols P152 and P300 were genotyped for *KIR* and *HLA* alleles using the Luminex platform. Linear regression was used to test the association between genotypes and baseline pre-ART HIV RNA, CD4^+^ lymphocyte count, and cognitive score, adjusting for age, race/ethnicity and study. The interaction between genetic markers and age was investigated. To account for multiple testing the false discovery rate (FDR) was controlled at 0.05.

**Results:**

Children with the KIR2DS4*ALL FULL LENGTH (KIR2DS4*AFL) allele had higher CD4^+^ lymphocyte counts. Among children ≤2 years of age, the KIR2DS4*AFL was associated with lower plasma HIV RNA and higher cognitive index scores. *KIR Cent2DS3/5_1* had lower CD4^+^ lymphocyte counts in children ≤2 years of age, while the presence of *Tel1*, *Tel2DS4_2*, *Tel2DS4_4*, *Tel8*, *Tel2DS4_6* had higher CD4^+^ lymphocyte counts in all children. Presence of *Cent2*, *Cent4 and Cent8* was associated with increased HIV RNA load in children ≤2 years. Presence of *KIR3DL1+Bw4* was associated with higher CD4^+^ lymphocyte counts in all children. Among children >2 years old, *KIR3DS1+Bw4-80I* was associated with higher plasma HIV RNA, and *Bw6/Bw6* was associated with lower plasma HIV RNA compared to children with *KIR3DS1+Bw4-80I*.

**Conclusions:**

Presented data show for the first time that specific *KIR* alleles independently or combined with HLA ligands are associated with HIV RNA and CD4^+^ lymphocyte counts in infected, antiretroviral naive children; and many of these effect estimates appear to be age dependent. These data support a role for specific *KIR* alleles in HIV pathogenesis in children.

## Introduction

Natural killer (NK) cells are key components of the innate immune system that act as the first line of defense and regulate antiviral immune responses [1]. NK cells mediate cytotoxicity and cytokine release via a large panel of activating and inhibitory receptors [2, 3]. Although human leukocyte antigen (*HLA*) gene products are fundamental to acquired immune responses, they are also important in innate immunity as ligands for the killer cell immunoglobulin-like receptors (KIRs) that modulate NK cell activity [4]. HLA class I molecules closely regulate KIR functions. Both the families of *KIR* and the *HLA* class I genes are extremely diverse suggesting that NK cell mediated innate immune responses are at least partly genetically predetermined [3;5]. KIRs are expressed on both T cells as well as NK cells and may inhibit or activate their function. *HLA* and *KIR* subtype combinations can mount unique innate immune responses against human immunodeficiency virus type-1 (HIV) infection [6]. *HLA* and *KIR* allele combinations can be both protective and deleterious against HIV-related disease progression [7;8] and can affect mother-to-child HIV transmission [9]. Independent and combined *KIR and HLA (KIR/HLA)* genotypes and haplotypes with an activating profile (presence of activating *KIRs* or absence of inhibitory *KIR*s or their respective HLA ligands) have been associated with HIV disease [10–17]. Effects of *KIR/HLA* alleles on HIV disease of children have not been previously studied. In the analyses presented here, we estimated the effects of *KIR* and *HLA* genotypes on plasma HIV RNA, CD4^+^ lymphocyte count and cognitive index score using a unique cohort of antiretroviral naïve HIV-infected children.

## Subjects and Methods

### Participants

Nine hundred and ninety three antiretroviral naïve children with symptomatic HIV infection from Pediatric AIDS Clinical Trial Group (PACTG) protocols P152 and P300 were included in the analyses [18;19]. P152 and P300 were multicenter, prospective, randomized, double blind, placebo controlled trials that assessed the efficacy of combination nucleoside reverse transcriptase inhibitor (NRTI) treatment regimens in symptomatic HIV-infected children in the United States prior to the availability of effective combination antiretroviral therapy. Important eligibility criteria included children of an age range of 3 months to 18 years with symptomatic HIV infection for P152 [18], and an age range of 42 days to 15 years with symptomatic HIV infection for P300 [19]. In these two protocols, CD4^+^ lymphocyte count, plasma HIV RNA and the cognitive score were measured at entry prior to initiation of therapy [18;19].

### Methods

Viral load was assayed with the Roche Amplicor quantitative RNA PCR method (limit of detection 400 copies/mL; 2.6 log_10_RNA copies/mL). The age appropriate neuropsychologic evaluations [20] included Bayley (42 days to 36 months) [21]; Wechsler Preschool and Primary Scales of Intelligence-Revised (WPPSI-R, 36 months to 6 years) [22]; Wechsler Intelligence Scale for Children: Revised (WISC-R III, 6 years to 17 years) [23] and Wechsler Adult Intelligence Scale: Revised (WAIS–R, >17 years) [24] for P300. P152 used Bayley scales, McCarthy [25] scales, WISC- R and WAIS–R as age appropriate. All cognitive scores were standardized (mean = 100, SD = 16). Children having a cognitive score below 70 are typically considered impaired. Studies followed the human experimentation guidelines of the US Department of Health and Human Services. The University of California San Diego Institutional Review Board has approved this study. Parents or legal guardians provided written informed consent to participate in these studies. Written informed consent had to be signed prior to participation in the studies. Each participating site was required to have Institutional Review Board approval prior to initiating the studies at their site.

### Genotyping

Stored DNA samples from the 993 children were assayed for *KIR* alleles using LIFECODES KIR-SSO TYPING KIT on the Luminex platform (Kashi Clinical Laboratories, Inc. Portland, OR). Total genomic DNA was extracted from peripheral blood mononuclear cells (PBMCs) using QIAamp DNA Blood Mini Kit (Qiagen, Carlsbad, CA). Whole genome amplification of DNA was done using Qiagen WGA kits [26]. The following KIR allelic variants were genotyped: *2DL1*, 2DL2 (2DL2*001/2/3/5, 2DL2*004), *2DL3*, *2DL4*, *2DL5*, *2DS1*, *2DS2*, *2DS3*, 2DS4* ALL FULL LENGTH (AFL), 2DS4*deletion exon 5 (Ex.5), 2DS4*full length exon 5 (Ex.5), *2DS5*, *3DL1*, *3DL2*, *3DL3*, *3DS1*, 3DS1*49N, *2DP1*, and 3DP1. KIR2DS4*AFL probe confirmed the presence of the full length *KIR2DS4* gene, while the subsequent two probes further characterized the exon 5 such that KIR2DS4*deletion Ex.5 represented a deletion in exon 5 while KIR2DS4*full length Ex.5 represented no deletion in exon 5. Pseudogenes (*KIR3DP1* and *KIR2DP1*) that do not code functional KIR receptors were excluded from the analyses. Delimiting alleles *KIR2DL4* and *KIR3DL3* were positive in all children and were therefore excluded from the analyses.

The *KIR* locus on chromosome 19 was split into the centromeric (Cent) and telomeric (Tel) regions and analyzed as described earlier [27]. KIR centromeric alleles (Cent 1–9) included *2DS2*, *2DL2*, *2DL3*, *2DL1*; telomeric alleles (Tel 1–8) included *3DS1*, *2DS1*, *3DL1*, *2DS4* and combined centromeric and telomeric (Cent/Tel) alleles included *2DL5*, *2DS3*, and *2DS5*. The Cent/Tel KIR motif with *KIR2DL5* variants are grouped in 13 different loci, the Cent KIR motif with *KIR2DL1* and *KIR2DS3/KIR2DS5* genes were grouped in 8 different loci (Cent-*2DS3/5 1–8*), and the Tel *KIR* motif with *KIR2DS4* Full/del variant subtypes were grouped in 8 different loci (Tel-*2DS4* 1–8). Activating KIR alleles included *2DS4*, *2DS1*, *2DS2*, *2DS3/2DS5*, *3DS1* and inhibiting alleles include *2DL5A*, *3DL1*, *2DL1*, *2DL5B*, *2DL2/2DL3*. *KIR2DL4* encodes a receptor that has both inhibitory [28] and activating functions [29;30]. HLA genotyping was performed using Lifecodes HLA SSO (Immuncor, Norcross, GA) by multiplexing using Luminex 100 platform (Luminex Corp, Austin, TX) at Tepnel Lifecodes Corporation (Stamford, CT) for *HLA-A*, *B*, *C*, *HLA DRB* alleles, as previously described [31]. For considering HLA-C molecules as ligands of NK cells, all *HLA-C* alleles can be grouped in two major KIR epitopes, HLA-C*01/*03/*07/*08/*12/*14/*16 alleles as *HLA-C1* group and HLA-C*02/*04/*05/*06/*15/*17/*18 alleles as *HLA-C2* group [32]. HLA-C1 molecules are ligands for inhibitory KIR2DL2/3 and activating KIR2DS2 receptors; and HLA-C2 molecules are ligands for inhibitory KIR2DL1 and activating KIR2DS1 receptors [33, 34].

### Statistical methods

Individual KIR genotypes and haplotypes [35] were analyzed for their association with HIV disease. Additionally, KIR and HLA alleles were analyzed independently and in combination (including previously reported KIR3DL1, KIR3DS1 with Bw4, Bw4-80I or Bw4-80T alleles) [6–8] for their effects on immunological, virological and neurocognitive outcomes. An indicator variable was created to indicate the joint presence of specific KIR and HLA alleles. In HLA-Bw6/Bw6 individuals, it does not matter which KIR3DL1 subtype is present because the ligand Bw4 is absent, and KIR3DL1 molecule is non-functional in these individuals. The Bw6/Bw6 group was also used as a control group for the analysis of the KIR subtypes.

Multivariable linear regression was used to test the association between the *KIR* and *HLA* allelic variants, and three baseline outcome measures: HIV RNA load, CD4^+^ lymphocyte count, and cognitive score. In order to correct for heterogeneity of variance, the robust variance estimator was used [36]. As children ≤2 years of age often have more rapid disease progression than those older than 2 years and have more immature immune systems, it was hypothesized that the effects of host genetics on HIV disease may vary with age. Therefore, the interaction between each genetic marker and age group (age ≤2 years and >2 years) was investigated. For genetic markers with a marginally significant (p <0.1) genotype by age (age ≤2 years and >2 years) interaction, regression models were fit to each age group separately. Potential confounders that were included in the adjusted analyses were determined *a-priori* and included age, race/ethnicity and study (P152 vs. P300).

To control the false discovery rate (FDR), we used methods developed by Benjamini and Hochberg [37] and evaluated the results with the FDR value set at 0.1, as well as 0.05. All genetic associations with a p-value <0.05 in adjusted models were included in the summary tables along with the corresponding 95% confidence intervals (CI). Associations with an FDR <0.05 were considered to be statistically significant, while those with an FDR between 0.05 and 0.1 were considered to be marginally significant.

## Results

### Baseline characteristics

Of the 993 antiretroviral naïve children with symptomatic HIV infection, 430 (43%) were from P152 and 563 (57%) from P300; 453 (46%) were male. The median age was 2.3 years with 605 (61%) identified as Black, 245 (25%) Hispanic, 127 (13%) White and 16 (2%) as ‘Others’ race/ethnicity. Of the 986 subjects with baseline CD4^+^ lymphocyte counts, the median baseline CD4^+^ lymphocyte count was 778 count/mm^3^; 825 subjects had baseline HIV RNA data with a median baseline log_10_ RNA of 5.14. Of the 935 subjects with available baseline cognitive scores, the median baseline score was 83. Detailed characteristics of this population are provided in **Table 1**.

**Table 1:** Baseline characteristics.

Characteristics		Total (N = 993)
Gender	Male	453 (46%)
	Female	540 (54%)
Study	152	430 (43%)
	300	563 (57%)
Race	Black	605 (61%)
	Hispanic	245 (25%)
	White	127 (13%)
	Other	16 (2%)
Age (years)	N	993
	Mean (s.d.)	3.77 (3.84)
	Median (Q1, Q3)	2.31 (0.84, 5.56)
	(0, 2]	460 (46%)
	(2, 18)	533 (54%)
Baseline CD4^+^ lymphocyte count (cells/mm^3^)	N	986
	Mean (s.d.)	981.31 (838.84)
	Median (Q1, Q3)	777.96 (412.45, 1,318.83)
Baseline CD4^+^ lymphocyte percent	Mean (s.d.)	23.71 (11.98)
	Median (Q1, Q3)	23.92 (16.00, 31.00)
Baseline plasma HIV RNA (copies/ml)	N	825
	Mean (s.d.)	868,428 (2,465,255)
	Median (Q1, Q3)	139,476 (33,000,510,000)
Baseline log_10_ plasma HIV RNA (copies/ml)	Mean (s.d.)	5.10 (0.94)
	Median (Q1, Q3)	5.14
Baseline cognitive score	N	935
	Mean (s.d.)	81.73 (17.63)
	Median (Q1, Q3)	83

Distribution of *KIR* alleles, centromeric (*Cent*), telomeric (*Tel*) and combined *Cent/Tel* alleles and combined *KIR/HLA* alleles in the studied cohort are listed in **Tables 2, 3 and 4**.

**Table 2:** Frequency of *KIR* alleles.

*KIR* Genotypes	N (% Positive)
*KIR2DL1*	966 (97%)
*KIR2DL3*	719 (72%)
*KIR2DL4*	993 (100%)
*KIR2DL5*	547 (55%)
KIR2DL2*004	31 (3%)
KIR2DL2*001/2/3/5	544 (55%)
*KIR2DP1*	964 (97%)
*KIR2DS1*	284 (29%)
*KIR2DS2*	527 (53%)
*KIR2DS3*	290 (29%)
*KIR2DS5*	344 (35%)
KIR 2DS4*all full length	957 (96%)
KIR 2DS4*deletion ex5	660 (66%)
KIR 2DS4*full length ex5	598 (60%)
*KIR3DL1*	936 (94%)
*KIR3DL2*	992 (99%)
*KIR3DL3*	993 (100%)
*KIR3DP1*	993 (100%)
*KIR3DS1*	224 (23%)
*KIR3DS1/49*	6 (1%)

**Table 3:** Frequency of the *KIR* centromeric/telomeric alleles.

Centromeric/Telomeric alleles*	N (% Positive)
*Cent1*: *2DL1/2DL3*	719 (72%)
*Cent2*: *2DL1/2DL3/2DL2/2DS2*	323 (33%)
*Cent3*: *2DL1/2DL3/2DL2*	351 (35%)
*Cent4*: *2DL1/2DL3/2DS2*	329 (33%)
*Cent5*: *2DL1/2DL2*	535 (54%)
*Cent6*: *2DL1/2DL2/2DS2*	494 (50%)
*Cent7*: *2DL2/2DS2*	520 (52%)
*Cent8*: *2DL2/2DL3/2DS2*	323 (33%)
*Cent9*: *2DL3*	719 (72%)
*Cent-2DS3/5(1)*: *2DS3/2DS5/2DL1*	89 (9%)
*Cent-2DS3/5(2)*: *2DS5/2DL1*	330 (33%)
*Cent-2DS3/5(3)*: *2DS3/2DL1*	289 (29%)
*Cent-2DS3/5(4)*: *2DL1*	966 (97%)
*Cent-2DS3/5(5)*: *2DS3/2DS5*	89 (9%)
*Cent-2DS3/5(6)*: *2DS5*	344 (35%)
*Cent-2DS3/5(7) or Cent/Tel4*: *2DS3*	290 (29%)
*Cent-2DS3/5(8)*: *none of 2DS3/2DS5/2DL1*	12 (1%)
*Tel1*: *2DS4_ALL_FULL_LENGTH/3DL1*	924 (93%)
*Tel2*: *2DS4_ALL_FULL_LENGTH/3DL1/2DS1/3DS1*	174 (18%)
*Tel3*: *2DS4_ALL_FULL_LENGTH/3DL1/2DS1*	238 (24%)
*Tel4*: *2DS4_ALL_FULL_LENGTH/3DL1/3DS1*	190 (19%)
*Tel5*: *2DS4_ALL_FULL_LENGTH/3DS1/2DS1*	179 (18%)
*Tel6*: *3DS1/2DS1*	207 (21%)
*Tel7*: *2DS4_ALL_FULL_LENGTH*	957 (96%)
*Tel8 or Tel-2DS4(4)*: *3DL1*	936 (94%)
*Tel-2DS4(1)*: *2DS4_AFL+Del/3DL1*	637 (64%)
*Tel-2DS4(2)*: *2DS4_AFL/3DL1*	924 (93%)
*Tel-2DS4(3)*: *2DS4_ALL_Del/3DL1*	637 (64%)
*Tel-2DS4(5)*: *2DS4_ALL_FULL_LENGTH+Del*	660 (66%)
*Tel-2DS4(6)*: *2DS4_ALL_FULL_LENGTH*	957 (96%)
*Tel-2DS4(7)*: *2DS4_Del*	660 (66%)
*Cent/Tel1*: *2DS3/2DS5/2DL5*	89 (9%)
*Cent/Tel2*: *2DS5/2DL5*	331 (33%)
*Cent/Tel3*: *2DS3/2DL5*	290 (29%)
*Cent/Tel5*: *2DL5*	547 (55%)
*Cent/Tel6*: *none of 2DS3/2DS5/2DL5*	433 (44%)

*9 KIR centromeric alleles (Cent 1–9) included *2DS2*, *2DL1*, *2DL2*, *2DL3*; 8 telomeric alleles (Tel 1–8) included *3DS1*, *2DS1*, *3DL1*, *2DS4* and combined centromeric and telomeric (Cent/Tel) alleles included *2DL5*, *2DS3*, and *2DS5*. The Cent allele with *KIR2DL1* and *KIR2DS3/KIR2DS5* genes were grouped in 8 different loci (Cent-*2DS3/5*, *1–8*), and the Tel allele with *KIR2DS4* Full/del variant subtypes were grouped in 8 different loci (Tel-*2DS4*, 1–8).

**Table 4:** Frequency of the *KIR/HLA* alleles.

*KIR/HLA* Genotypes	Genotype Analysis Combinations	N (%)
*Bw4/Bw6*	*Bw4/Bw4*	278 (29%)
	*Bw4/Bw6*	471 (49%)
	*Bw6/Bw6*	220 (23%)
*KIR3DL1+Bw4*	*3DL1+Bw4*	626 (63%)
	*Bw6/Bw6*	310 (31%)
	*Non-3DL1+Bw4*	57 (6%)
*KIR3DL1+Bw4-80I*	*3DL1+Bw4-80I*	398 (40%)
	*Bw6/Bw6*	310 (31%)
	*Non-3DL1+Bw4-80I*	285 (29%)
*KIR3DL1+Bw4-80T*	*3DL1+Bw4-80T*	229 (23%)
	*Bw6/Bw6*	310 (31%)
	*Non-3DL1+Bw4-80T*	454 (46%)
*KIR3DS1+Bw4*	*3DS1+Bw4*	143 (14%)
	*Bw6/Bw6*	310 (31%)
	*Non-3DS1+Bw4*	540 (54%)
*KIR3DS1+Bw4-80I*	*3DS1+Bw4-80I*	79 (8%)
	*Bw6/Bw6*	310 (31%)
	*Non-3DS1+Bw4-80I*	604 (61%)
*KIR3DL1+B27/B57/Bw4-80I/Bw4-80T*	*3DL1+B27/B57/Bw4-80I/Bw4-80T*	564 (57%)
	*Bw6/Bw6*	310 (31%)
	*Non-3DL1+B27/B57/Bw4-80I/Bw4-80T*	119 (12%)
*KIR3DL1+Bw4/Bw4-80I/Bw4-80T*	*3DL1+Bw4/Bw4-80I/Bw4-80T*	626 (63%)
	*Bw6/Bw6*	310 (31%)
	*Non-3DL1+Bw4/Bw4-80I/Bw4-80T*	57 (6%)

### Associations of KIR and HLA alleles with baseline CD4^+^ lymphocyte counts

Results of association of independent and combined *KIR* and *HLA* alleles with CD4^+^ lymphocyte counts are summarized in **Table 5**.

**Table 5:** Association of *KIR/HLA* alleles with baseline CD4^+^ lymphocyte count.

Characteristics						Unadjusted Analysis		Adjusted Analysis		
SNP Type	SNP	Age by Genotype Interaction	Age Group	Level	Count	Difference (LCI, UCI)	P-value	Difference (LCI,UCI)	P-value	FDR Significance
*KIR*	*KIR 2DL2_004*	0.44	All	Positive	31	-227(-428,-27)	0.0264	-204(-384,-25)	0.0259	-
				Negative (Ref)	955	989(935,1042)	.	1379(1278,1480)	.	
	*KIR 2DS4_AFL*	0.19	All	Positive	950	279(112,447)	0.0011	265(103,426)	0.0013	**
				Negative(Ref)	36	712 (554,871)	.	1122 (952,1293)		
	*KIR 3DL1 (Tel 8)*	0.91	All	Positive	929	227(42,412)	0.0160	218(49,386)	0.0113	*
				Negative(Ref)	57	767 (591,944)	.	1170(985,1355)	.	
*KIR*centromeric/telomeric	*Cent-2DS3/5(1):2DS3/2DS5/2DL1#*	0.08	(0, 2]	Positive	36	-406(-646,-166)	0.0009	-431(-676,-185)	0.0006	**
				Negative(Ref)	419	1426(1329,1523)	.	1600 (1373,1827)		
			(2, 18)	Positive	52	-113(-229,2)	0.0550	-105(-209,-0.4)	0.0491	-
				Negative(Ref)	479	639 (601,678)	.	1005(918,1091)	.	
	*Cent/Tel1:2DS3/2DS5/2DL5#*	0.08	(0, 2]	Positive	36	-406(-646,-166)	0.0009	-431(-676,-185)	0.0006	**
				Negative (Ref)	419	1426(1329,1523)	.	1600 (1373,1827)		
			(2, 18)	Positive	52	-113(-229,2)	0.0550	-105(-209,-0.4)	0.0491	-
				Negative (Ref)	479	639 (601,678)	.	1005(918,1091)	.	
	*Tel1 or Tel-2DS4(2)*:*2DS4_AFL/3DL1*	0.61	All	Positive	917	250(85,414)	0.0029	232(81,383)	0.0026	**
				Negative(Ref)	69	749 (595,904)	.	1161(994,1327)		
	*Tel-2DS4(4)*: *3DL1*	0.91	All	Positive	929	227(42,412)	0.0160	218(49,386)	0.0113	*
				Negative(Ref)	57	767 (591,944)	.	1170(985,1355)	.	
	*Tel7 or Tel-2DS4(6)*:*2DS4_AFL*	0.19	All	Positive	950	279(112,447)	0.0011	265(103,426)	0.0013	**
				Negative(Ref)	36	712 (554,871)	.	1122 (952,1293)		
*KIR/HLA*	*KIR3DL1+Bw4*	0.19	All	Non-3DL1+Bw4	60	-266(-413,-119)	0.0004	-190 (-331,-49)	0.0083	*
				Bw6/Bw6	312	-1(-113,111)	0.98	-22(-122,78)	0.67	-
				3DL1+Bw4(Ref)	620	995 (926,1064)	.	1389(1278,1500)	.	
	*KIR3DS1+Bw4-80I*	0.15	All	Non-3DS1+Bw4-80I	601	169(-2,340)	0.0531	166(9,324)	0.0386	-
				Bw6/Bw6	312	171(-8,351)	0.0608	141(-23,305)	0.924	-
				3DS1+Bw4-80I(Ref)	79	823(666,979)	.	1230(1064,1397)		
	*KIR3DL1+Bw4/Bw4-80I/Bw4-80T*	0.14	All	Non-3DL1+Bw4/Bw4-80I/Bw4-80T	60	-266(-413,-119)	0.0004	-190 (-331,-49)	0.0083	*
				Bw6/Bw6	312	-1(-113,111)	0.98	-22(-122,78)	0.67	-
				3DL1+Bw4/Bw4-80I/Bw4-80T(Ref)	620	995 (926,1064)	.	1389(1278,1500)		
	*Bw4/Bw6: 3DS1 #*	0.054	(2,18)	Bw6/Bw6	29	269(61,477)	0.0112	209 (26,393)	0.0253	-
				Bw4/Bw6	63	191(45,338)	0.0105	133(0.4,266)	0.494	-
				Bw4/Bw4(Ref)	34	462(362,562)	.	814(655,972)		

*Adjusted analyses adjusted for age, study, and race

Ref: Reference group

#: The SNPs with a ‘#’ had genotype by age group p-value <0.01.

-: Not significant at FDR = 0.1

*: Significant at FDR = 0.1

**: Significant at FDR = 0.05

Children with KIR2DS4*AFL (*Tel2DS4(6) or Tel 7*), had a higher CD4^+^ lymphocyte count compared to those without it (adjusted mean difference (β) = 265, CI (103, 426), p = 0.001, significant at an FDR = 0.05 (FDR≤0.05). A test for concordance of KIR2DS4*AFL and *KIR3DL1* alleles showed a strong linkage disequilibrium (kendall’s τ = 0.51, p<0.001).

Among the KIR centromeric and telomeric alleles the presence of *KIR2DS3/2DS5/2DL1* (*Cent2DS3/5(1)* was associated with a lower baseline CD4^+^ lymphocyte count (β = -431, CI (-676, -185); p = 0.0006, significant at FDR = 0.05) in children ≤2 years old. *KIR2DS3/2DS5/2DL5* (*Cent/Tel1)* was in complete linkage disequilibrium with *KIR2DS3/2DS5/2DL1 (Cent2DS3/5(1))* and showed the same associations. Presence of *KIR2DS4_ AFL /3DL1 (Tel2DS4(2) or Tel1)* was associated with higher CD4^+^ lymphocyte count (β = 232, CI (81, 383); p = 0.003, significant at FDR = 0.05) for the children of all ages.

Among the combined *KIR/HLA* alleles, the absence of *KIR3DL1* and *Bw4 (non-KIR3DL1+Bw4*) was associated with a lower CD4^+^ lymphocyte count compared to *KIR3DL1+Bw4* (β = -204, CI (-350,-59), p = 0.006; marginally significant at FDR = 0.1).

### Associations of KIR and HLA alleles with baseline HIV RNA load

Results of association of independent and combined KIR and HLA alleles with HIV RNA are summarized in **Table 6**.

**Table 6:** Association of *KIR/HLA* alleles with baseline HIV Log_10_RNA.

Characteristics						Unadjusted Analysis		Adjusted Analysis		
SNP Type	SNP	Age by Genotype Interaction	Age Group	Level	Count	Difference (LCI, UCI)	P-value	Difference (LCI, UCI)	P-value	Significant Controlling for FDR at 0.1
*KIR*	*KIR 2DS2* #	0.0190	(0, 2]	Positive	189	0.2(0.0,0.4)	0.0290	0.2(0.0,0.3)	0.0354	-
				Negative (Ref)	193	5.5(5.4,5.6)	.	6.2(6.0,6.4)		
	*KIR 2DS4_AFL* # *(Tel 7 orTel-2DS4_6*	0.0447	(0, 2]	Positive	372	-0.7(-1.2,-0.3)	0.0016	-0.6(-1.0,-0.2)	0.0055	*
				Negative (Ref)	10	6.3(5.9,6.8)	.	6.9(6.5,7.3)		
*KIR* centromeric/ telomeric	*Cent2*:*2DL1/2DL3/2DL2/2DS2* #	0.0043	(0, 2]	Positive	117	0.3(0.1,0.5)	0.0024	0.2(0.1,0.4)	0.0063	*
				Negative (Ref)	265	5.5(5.4,5.6)	.	6.3(6.1,6.5)		
	*Cent3*: *2DL1/2DL3/2DL2* #	0.0128	(0, 2]	Positive	127	0.2(0.1,0.4)	0.0090	0.2(0.0,0.4)	0.0118	-
				Negative (Ref)	255	5.5(5.4,5.6)	.	6.3(6.1,6.5)		
	*Cent4*: *2DL1/2DL3/2DS2* #	0.0033	(0, 2]	Positive	120	0.3(0.1,0.4)	0.0030	0.2(0.1,0.4)	0.0051	*
				Negative (Ref)	262	5.5(5.4,5.6)	.	6.3(6.1,6.5)		
	*Cent6*: *2DL1/2DL2/2DS2* #	0.0373	(0, 2]	Positive	179	0.2(0.0,0.4)	0.0274	0.2(0.0,0.3)	0.0319	-
				Negative (Ref)	203	5.5(5.4,5.6)	.	6.2(6.0,6.4)		
	*Cent7*: *2DL2/2DS2* #	0.0255	(0, 2]	Positive	186	0.2(0.0,0.4)	0.0255	0.2(0.0,0.3)	0.0410	-
				Negative (Ref)	196	5.5(5.4,5.6)	.	6.2(6.0,6.4)		
	*Cent8*: *2DL2/2DL3/2DS2* #	0.0043	(0, 2]	Positive	117	0.3(0.1,0.5)	0.0024	0.2(0.1,0.4)	0.0063	*
				Negative (Ref)	265	5.5(5.4,5.6)	.	6.3(6.1,6.5)		
*KIR/HLA*	*KIR3DS1+Bw4-80I* #	0.0790	(2, 18)	Non-3DS1+Bw4-80I	267	-0.4(-0.6,-0.1)	0.0015	-0.4(-0.6,-0.1)	0.0008	*
				Bw6/Bw6	143	-0.4(-0.6,-0.1)	0.0029	-0.4(-0.6,-0.2)	0.0019	*
				3DS1+Bw4-80I(Ref)	38	5.0(4.8,5.2)	.	4.8(4.6,5.1)		

Adjusted analyses adjusted for age, study, and race

Ref: Reference group

#: The SNPs with a ‘#’ had genotype by age group p-value <0.01.

-: Not significant at FDR = 0.1

*: Significant at FDR = 0.1

In children ≤2 years old, the presence of KIR-2DS4* AFL, (*Tel2DS4(6)* or Tel7) was associated with lower log_10_ viral RNA load compared to those without it (β = -0.6, CI (-1.0, -0.2), p = 0.006; significant at FDR <0.05) which is in agreement with the higher CD4^+^ lymphocyte count observed above. This decrease in HIV RNA was not significant in the older age cohort, > 2 years old (FDR >0.1).

Among the *KIR* centromeric and telomeric alleles, the presence of *KIR2DL1/2DL3/2DL2/2DS2 (Cent2* or *Cent8)* was associated with higher HIV RNA load (β = 0.2, CI (0.1, 0.4); p = 0.006, marginally significant at FDR = 0.1) in children ≤ 2 years old. The presence of *KIR2DL1/2DL3/2DS2 (Cent4*) was associated with higher HIV RNA load (β = 0.2, CI (0.1, 0.4); p = 0.005, marginally significant at FDR = 0.1) in ≤ 2 year old children.

Among the combined *KIR/HLA alleles*, the absence of *KIR3DS1* and *Bw4-80I (non-KIR3DS1+Bw4-80I)* was associated with lower HIV RNA load compared to *3DS1+Bw4-80I* (β = -0.4, CI (-0.6,-0.1), p = 0.0014, significant at FDR <0.05). Also, the presence of *Bw6/Bw6* was associated with lower viral load compared to *3DS1+Bw4-80I* (β = 0.4, CI (-0.6,-0.2); p = 0.0009, significant at FDR <0.05). These estimated differences remained significant after the FDR adjustment.

No statistically significant associations were observed for any of the combined *KIR/HLA* alleles on the baseline cognitive score. Furthermore, no combination of *HLA-C1* or *C2* alleles with *KIR* alleles were significantly associated with the baseline CD4 count, logRNA viral load or cognitive score.

## Discussion

Natural killer cells modulate antiviral immune response [38] by mediating the KIR mediated lysis of the targeted infected cells [10–17]. Through the release of various cytokines, a strong adaptive immune response is activated that leads to T cell proliferation and a reduction in viral replication [39]. Functions of NK cells are regulated by the activating or inhibiting KIRs and their HLA class I ligands [4]. Thus, the presence of *KIR* alleles coding specific NK receptors and *HLA* Class I ligand alleles for NK cell function can alter the anti-HIV innate immune response in infected children. In the research presented, we tested the association of the specific KIR alleles independently or in combination with ligand *HLA* class I alleles with the HIV-related disease status markers in children.

First, we estimated the effects of independent *KIR* alleles on HIV disease. We found that the presence of the NK cell activating allele, KIR2DS4*AFL was associated with a higher CD4^+^ lymphocyte count and lower viral RNA loads in children ≤2 years old, as well as an increase in baseline cognitive score. *KIR2DS4* is an activating gene for NK function. Hence, the presence of *KIR2DS4* is expected to be associated with a functional NK response and protective effects against HIV infection and disease. Contrary to our findings, a study conducted in antiretroviral naïve adults has recently shown that *KIR2DS4* promotes HIV pathogenesis [40]. Reasons for the difference in our findings are unclear. It is possible that in adults, the adaptive immune response via HLA-HIV peptide-CD8 interactions predominates over the *KIR* mediated innate immune response observed in children.

The presence of inhibiting allele KIR2DL2*004 was associated with lower CD4^+^ counts compared to those without it. This effect of *KIR2DL2* has been reported in adults where the presence of *KIR2DL2* was also shown to be associated with a more rapid rate of CD4^+^ lymphocyte decline due to inhibition of NK cell function [17].

Similar to *KIR2DL2 allele*, the presence of the *KIR2DS2* allele has been shown to be associated with a more rapid rate of CD4^+^ T lymphocyte decline [17]. Consistent with these findings, our study showed that the presence of *KIR2DS2* allele was associated with higher plasma HIV RNA in children ≤2 years old.

A unique aspect of our study was the evaluation of the association of *KIR* alleles with HIV-related central nervous system (CNS) impairment. Of note, although a few *KIR* alleles had a p-value <0.05 for the cognitive score analyses, none retained significance after controlling for FDR. In a simian immunodeficiency virus (SIV) model of encephalitis in macaques, animals lacking strong NK cell responses developed more severe CNS lesions than those with robust responses [41]. *In vivo*, both macaque and human cells showed that NK cells mediated anti-SIV and anti-HIV cytolytic effects directed against the envelope protein. Hence, NK cells recognize and lyse cells expressing SIV and HIV antigens suggesting that NK cells affect HIV related CNS disease [41]. Thus, activating and inhibiting *KIR* alleles can modulate these CNS effects against HIV as observed in our studies.

Second, we investigated the effects of *KIR* centromeric and telomeric alleles on the HIV related disease in children. Among these, the presence of centromeric allele *2DS3/2DS5/2DL1 [Cent2DS3/5(1)]*, *2DL1/2DL3/2DL2 (Cent3)*, *2DL1/2DL3/2DS2 (Cent4) or 2DL2/2DL3/2DS2 (Cent8)* was associated with higher HIV RNA load in children ≤ 2 years old probably because of the predominant presence of inhibiting KIR molecules that inhibited NK cell function. Among the *KIR* telomeric alleles, the presence of *2DS4_AFL/3DL1 [Tel-2DS4(2)] or 2DS4AFL/3DL1 (Tel1))*, *3DL1 [Tel2DS4(4) or Tel8]* and *2DS4AFL [Tel2DS4(6) or Tel 7]* was associated with higher CD4^+^ lymphocytes count and lower viral RNA load in the whole cohort because of the preponderance of stimulating KIR molecules that activated NK cells for killing of HIV infected cells. Significant age and genotype interactions observed in our study may suggest an age dependent maturation of the adaptive immune response reflected in *KIR/HLA* mediated NK cell response.

Finally, we tested the presence of combined *KIR* and *HLA* alleles on HIV disease in children. *KIR* and *HLA* class I alleles have been shown to act both independently and synergistically to modify HIV disease progression in adults [17]. *HLA-Bw4* molecules with isoleucine at position 80 (*Bw4-80I*) are ligands for inhibitory *KIR3DL1* receptors and *Bw4-80Ile*. Combined with the activating *KIR3DS1* allele, these have been found to be associated with delayed progression to AIDS [11]. In our study, the absence of *KIR3DL1* and *Bw4* (non-*KIR3DL1+Bw4*) was associated with a lower CD4^+^ lymphocyte count. These results are in concordance with those in adults where the presence of *KIR3DL1* and *Bw4* was associated with slower HIV disease progression [6]. NK cells kill their HIV-infected target cells in a receptor ligand-specific manner that involved activating KIR3DS1 and its putative ligand HLA-Bw4-80I [42]. However, in the current study, different to other studies in adults [10], the absence of *KIR3DS1+Bw4-80I* was associated with lower HIV RNA load compared to those with *Bw6/Bw6* group. Reasons for these differences are not clear but may reflect a different NK cell mediated innate immune response in children compared to adults. This may also be explained by the unique nature of our cohort (approximately 61% African-American), wherein the frequencies and protective effects of HLA-B alleles on HIV disease progression differs from Caucasian cohorts.

In an earlier study in adults, the frequency of the *KIR3DS1 (3DS1/3DL1)-Bw4* combination was significantly higher in highly exposed and persistently seronegative patients versus discordant couples [6;12]. Higher frequency of *KIR3DS1/3DL1* heterozygotes and HLA-*Bw4-80I* has been associated with long-term non-progressors [10]. Consistent with these findings, our study showed that the presence of *KIR3DS1* and *Bw4-80I* was associated with higher CD4^+^ lymphocyte counts and lower HIV RNA.

Due to the presence of different *KIR* and *HLA* alleles, the varied expression of KIRs on different NK cells and CD8^+^ T lymphocytes potentially generate selective antiviral responses. For example, *HLA-B* alleles encode a peptide epitope sequence that controls allele-specific interactions with the inhibitory *KIR3DL1* allele. HIV infected cells that may avoid immunosurveillance by downregulating the HLA-B expression, are killed by NK cells upon the loss of inhibitory NK receptor signals such as *KIR3DL1*. Thus, NK receptors directly participate in the adaptive immune response due to the expression of KIRs on CD8^+^ T cells [43;44].

An association of *KIR/HLA* alleles has been reported with the risk for HIV mother-to-child-transmission in two studies [45;46]. In these studies, the presence of *KIR2DS4* allelic variants had differential effects on *in utero* and intrapartum transmission. Additionally, a strong association has been observed with maternal *HLA-B* alleles independent of viral load. This finding implicates innate immune mechanisms via NK receptor KIR3DL1 that are triggered by a decrease in the expression of *HLA* class I molecules [47]. A decreased *HLA*-*B* expression on infected cells removes the inhibitory signal by *KIR3DL1* and lowers the threshold for CD8^+^ T-cell activation by viral peptides enhancing the adaptive CD8^+^ T-cell anti-HIV response. Furthermore, a blockade of NK cell inhibiting KIR molecules can also improve the anti-HIV-1 activity of NK cells [48].

The presence of *HLA-C1* or *C2* alleles in combination with *KIR2DL* or *KIR2DS* alleles has been reported to be associated with HIV disease [49;50]; however we did not observe it in the children cohort. Reasons for these results are not clear but as noted above, these may reflect a difference in the maturity of NK cell mediated innate immune response in children compared to adults and the unique nature of our predominant African-American cohort.

In summary, our study has shown that *KIR* alleles are associated with altered HIV disease pathogenesis in children independently and in combination with *HLA* class I ligands. In general, effects of *KIR* alleles on HIV disease in children follow the pattern observed in adults. Additionally, there was an age dependent association of *KIR* alleles with HIV disease observed particularly in younger children suggesting an effect of maturation of innate and adaptive immune responses. These studies will help guide the development of *KIR/HLA* based therapeutic targets against HIV disease.
